# Comparisons of infant *Escherichia coli* isolates link genomic profiles with adaptation to the ecological niche

**DOI:** 10.1186/1471-2164-14-81

**Published:** 2013-02-05

**Authors:** Eric J de Muinck, Karin Lagesen, Jan Egil Afset, Xavier Didelot, Kjersti S Rønningen, Knut Rudi, Nils Chr Stenseth, Pål Trosvik

**Affiliations:** 1 Centre for Ecological and Evolutionary Synthesis (CEES), Department of Biosciences, University of Oslo, PO Box, 0316 Oslo, 1066, Norway; 2 Division of Epidemiology, Norwegian Institute of Public Health, PO Box, 0456 Oslo, 4404, Norway; 3 NOFIMA - The Norwegian Institute of Food, Fisheries and Aquaculture Research, PO Box, 1430 Ås, 210, Norway; 4 Department of Laboratory Medicine, Children’s and Women’s Health, Faculty of Medicine, Norwegian University of Science and Technology, PO Box, 7491 Trondheim, 8905, Norway; 5 Department of Medical Microbiology, St Olavs Hospital, PO Box, 7006 Trondheim, 3250, Norway; 6 Department of Infectious Disease Epidemiology, Imperial College London, St Mary’s Campus, Norfolk Place, London, W2 1PG, UK; 7 Department of Pediatric Research, Oslo University Hospital, Rikshospitalet, PO Box, 0424 Oslo, 4905, Norway; 8 Department of Chemistry, Biotechnology and Food Science, University of Life Sciences, PO Box, 1432 Ås, 5003, Norway

**Keywords:** *Escherichia coli*, Comparative genomics, Infant gut, Commensal, Pathogen, Generation time, Codon usage bias

## Abstract

**Background:**

Despite being one of the most intensely studied model organisms, many questions still remain about the evolutionary biology and ecology of *Escherichia coli*. An important step toward achieving a more complete understanding of *E.coli* biology entails elucidating relationships between gene content and adaptation to the ecological niche.

**Results:**

Here, we present genome comparisons of 16 *E.coli* strains that represent commensals and pathogens isolated from infants during a specific time period in Trondheim, Norway. Using differential gene content, we characterized enrichment profiles of the collection of strains relating to phylogeny, early vs. late colonization, pathogenicity and growth rate. We found clear gene content distinctions relating to the various grouping criteria. We also found that different categories of strains use different genetic elements for similar biological processes. The sequenced genomes included two pairs of strains where each pair was isolated from the same infant at different time points. One pair, in which the strains were isolated four months apart, showed maintenance of an early colonizer genome profile but also gene content and codon usage changes toward the late colonizer profile. Lastly, we placed our sequenced isolates into a broader genomic context by comparing them with 25 published *E.coli* genomes that represent a variety of pathotypes and commensal strains. This analysis demonstrated the importance of geography in shaping strain level gene content profiles.

**Conclusions:**

Our results indicate a general pattern where alternative genetic pathways lead toward a consistent ecological role for *E.coli* as a species. Within this framework however, we saw selection shaping the coding repertoire of *E.coli* strains toward distinct ecotypes with different phenotypic properties.

## Background

Awareness of the importance of the gut microbial colonization for human health is growing as numerous links with a multitude of diseases are being discovered [1]. Recent advances in sequencing technology have generated massive amounts of data, but much remains to be understood about the processes important for maintaining a healthy community structure. *E.coli*, as well as being a much studied model organism, is an important and ubiquitous member of the human gut microbial community. Although *E.coli* constitutes only a small fraction of the total gastrointestinal microbiota, it has a wide spectrum of potential interactions with the human host, ranging from probiotic to commensal and on to pathogenic [2].

As one of the most intensely studied organisms, much genomic information on this species has already been collected. Genbank has cataloged 60 complete chromosomal genomes and 346 draft genomes (at the time of writing). However, less sequencing effort has been directed toward truly commensal *E.coli* strains, relative to pathogenic isolates and derivatives of popular laboratory strains. Previous comparative analysis of the genome sequences of 61 isolates has helped develop a new view of the *E.coli* genetic landscape which highlights diversity at the genome level [3]. A typical *E.coli* strain carries between 4,000 and 5,500 genes. On average, an *E.coli* strain will share about 40% of these with all other members of the species, while the remainder forms part of the pan-genome [4,5]. Following these approaches, differential gene content between strains is thought to subdivide *E.coli* into ecological classes that may be more biologically informative than traditional phylogenetic categorization based for example on Multi-Locus Sequence Typing (MLST). Use of full genomes and subsequent gene-content profiling has thus become important for understanding the role of genome contents for defining a realized ecological niche [6].

This work is a continuation of a deep characterization of *E.coli* strains isolated from a cohort of infants and their mothers in Trondheim, Norway. The original study design was a nested case–control format created to examine the impact of whole gut microbial colonization on the development of atopic disease [7,8]. In this original characterization of the cohort, qPCR was used to identify and quantify the microbial fecal composition of several classes of bacteria and these data were matched with cytokine profile development. From this, it was observed that early *E.coli* colonization was linked to protection from atopy and the mother was found to be a likely source of infant colonization [9]. We have previously characterized the *E.coli* colonization pattern of a sub-cohort of this larger study, 85 infants and their mothers, and found limits on the diversity of strains and further evidence of transmission from the mothers to the infants [10]. This same study then placed these strains into a phylogenetic context of the wider *E.coli* diversity.

Here, we built upon these earlier observations using whole genome sequencing. We compare the genomic content of strains with different phylogenetic, pathogenic vs. commensal, growth rate and early vs. late colonization characteristics in order to determine enrichment profiles that may explain these ecological traits. The signatures that were observed can be used for further investigations into genotype-phenotype mapping within the context of ecological adaptation, and for investigating the role of the many hypothetical proteins that we found differentiating the groups. The collection of strains that were used for this analysis offer insight into a temporally and geographically coherent population of gut colonizing *E.coli*, with additional context afforded by our previous characterizations of these strains [10]. Methodological challenges that were addressed included developing a strategy for compensating for incomplete assembly, small sequencing errors, and potential loss of genetic information derived from genomes sequenced by 454 single-end shotgun sequencing. Dealing with incompletely assembled draft genomes, as we have done, may become less problematic for single isolate analysis as assembly algorithms and sequencing technologies progress. However, costs may hinder coverage for large collections of isolates and also for complex samples such as the soil or mammalian gut, which at minimum contains several hundred genomes [11,12].

## Results

### Methodological challenges

454 sequencing of the 16 genomes yielded coverage levels, the median number of times that a specific genomic position is included in a sequencing read, ranging from 7.55 to 20.1 (Table 1). Good coverage is crucial to aid assembly of the reads into as few contigs as possible. This was indicated in our data by the significant decrease of contig numbers as the median coverage depth increased (R^2^ = 0.69, p < 0.0001, Additional file 1: Figure S1A). However, there was a plateau at about 13× coverage, above which that trend subsided. We also found a strong positive correlation between the number of annotated genes (see Materials and Methods) per base in a genome and the number of contigs in the assembly (R^2^ = 0.52, p = 0.0017) (Additional file 1: Figure S1B). Furthermore, there was an even stronger trend for mean annotated gene length to decline as assemblies became more fragmented (R^2^ = 0.94, p < 0.000001, Additional file 1: Figure S1C). This indicated that partial gene sequences were more often retrieved from the more fragmented assemblies. The main cause of this phenomenon was genes being split onto two different contigs due to reduced coverage at contig edges in low read depth assemblies (Additional file 1: Figure S1D), or small sequencing errors, usually in homopolymer tracts (a known shortcoming of pyrosequencing), producing spurious frame-shifts in the coding sequence. Both of these causes can result in coding sequences being un-annotated by RAST. To circumvent this issue, we applied an additional gene recovery step (see Materials and Methods) which resulted in a positive relationship between the number of BLAST hits retrieved and the number of contigs in an assembly (R^2^ = 0.57, p = 0.0008, Additional file 1: Figure S1E), with a total of 8,322 genes being recovered from all the strains combined. Following this curation of the genome annotations, we re-examined the bias in the relationship between the number of annotated genes per base and the number of contigs and found that the pre-treatment bias had been completely removed (R^2^ = 0.0001, p = 0.97, Additional file 1: Figure S1F). This information, and the continued strong correlation (R^2^ = 0.92) between the number of gene families and genome size (Additional file 1: Figure S1G) suggest that the updated annotations were correct. One outlier (JEA297p) showed a different gene density than the other strains, and will be discussed below.

**Table 1:** List of strains used in this study with corresponding genome information

**ID (alt. ID)**	**child number**	**Clinical condition**	**Age at Sampling**	**Phylogenetic group**	**Nr. contigs**	**Median coverage**	**Colonization Category**	**Accession Accession Number**
EDM1c	1360	Healthy	10 days	B2	712	7,55	early	EMBL:ERS155053
EDM3c	1360	Healthy	1 year	B1	306	12,4	late	EMBL:ERS155049
EDM16c	1870	Healthy	7 days	B1	204	13,75	early	EMBL:ERS155051
EDM70c	1997	Healthy	10 days	B2	562	8,5	early	EMBL:ERS155055
EDM49c	1891	Healthy	4 days	B2	163	14,5	early	EMBL:ERS155056
EDM101c	1891	Healthy	11 days	B2	169	16	early	EMBL:ERS155057
EDM106c	123	Healthy	4 days	B2	585	8	early	EMBL:ERS155058
EDM116c	123	Healthy	1 year	A	864	8,2	late	EMBL:ERS155052
EDM123c	1360	Healthy	4 months	B2	669	8,5	late	EMBL:ERS155054
EDM530c	123	Healthy	2 years	NA	198	17,5	late	EMBL:ERS155050
JEA117c (Trh9)*	117c	Healthy	1 year	B2	284	10,1	late	EMBL:ERS178156
JEA242p (Trh52)*	242p	Diarrhoea	3 years	B2	140	13,2	NC	EMBL:ERS178157
JEA297p (Trh58)*	297p	Diarrhoea	2 years	D	521	11,1	NC	EMBL:ERS178158
JEA179p (Trh39)*	179p	Diarrhoea	4 years	B1	848	7,8	NC	EMBL:ERS178159
JEA160c (Trh12)*	160c	Healthy	2 years	A	188	20,1	late	EMBL:ERS178160
JEA124p (Trh29)*	124p	Diarrhoea	2 years	A	800	8,9	NC	EMBL:ERS178161

c = commensal isolate, isolated from healthy child.

p = pathogenic isolate, isolated from child with diarrhoea.

* Isolation based on positive PCR analysis for intimin gene *eae.*

NC = not categorized.

The genome assembly statistics are results of Newbler *de novo* assembly. The colonization categorizations are the ones used for gene enrichment comparison between early and late colonizers. The 10 strains named EDM were isolated from samples originating from the IMPACT study [8,10,13]. JEA strains and alternative strain IDs are from [14].

### Phylogenetic and gene content comparisons

**Figure 1:**
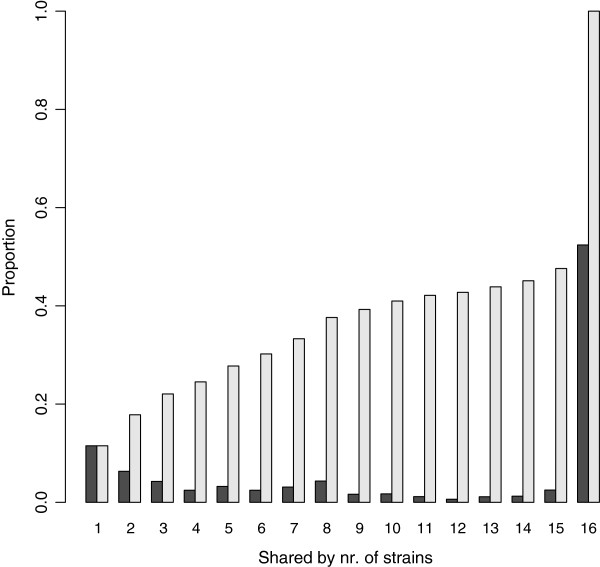
**Overview of relative and cumulative proportions of shared gene families as the number of included genomes increases.** Each pair of bars along the x-axis represents the relative (dark grey) and cumulative (light grey) proportions of gene families that are shared among the indicated number of strains. E.g. the first pair of bars indicates the proportion of gene families that were found in only one of the 16 strains, the second pair indicates the proportion shared by two of the 16 strains, while the final pair indicates the proportion of the pan-genome that is common to all 16 strains. All duplicated gene annotations were removed for this analysis. 11.5% of annotated genes are unique to one strain while 52.4% are common to all. The total number of gene families in the pan-genome is 6152.

**Figure 2:**
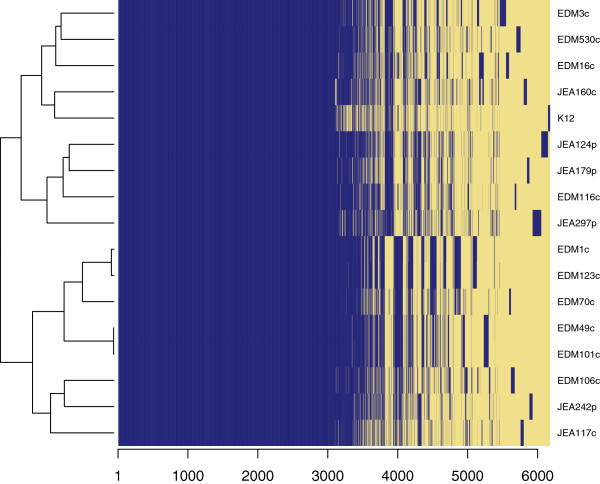
**Heat map of total gene content comparisons.** Gene presence is shown in blue and gene absence in yellow. The number of genes is depicted on the x-axis. Strains are listed in the order following hierarchical clustering created using a Manhattan distance matrix based on the gene presence/absence gene content matrix.

**Figure 3:**
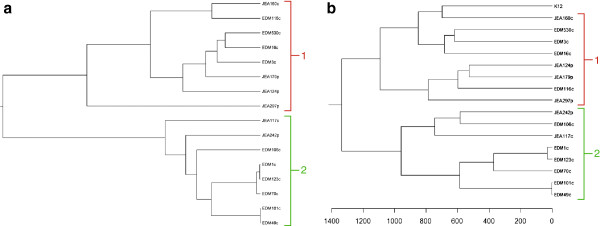
**Comparison of genome trees generated by core and pan-genomes. a**. The core genome phylogeny was created using ClonalFrame. **b**. The pan-genome tree was created using hierarchical clustering of a Manhattan distance matrix based on the gene presence/absence matrix. The scale below the pan-genome tree indicates Manhattan distances. Both methods separated the strains into two main clades (1 and 2).

Comparative analysis of gene content revealed that 52.4% of the genes are shared by our 16 genomes (Figure 1), representing a core genome of 3224 gene families. However, inclusion of strain MG1655 (K12) reduced the proportion of shared genes to 50.2%. This is higher than results reported in other studies [3,5] and could be attributed to the localized sampling of our *E.coli* population. The pan-genome of our 16 strains included 6,152 gene families. This number increased to 6,181 when K12 was included (Figure 2). The structure of the dendrograms generated from the genome collection were similar whether we used homology in the core genome or gene content differences to determine their relationships, with the deepest subdivision being between the clades denoted 1 and 2 (Figures 3A and 3B). Human-colonizing *E.coli* are generally grouped into the four phylogroups: A, B1, B2 and D [2]. Here, clade 2 contained all the strains previously categorized into the B2 phylogroup, whereas members of clade 1 belonged to phylogroups A, B1 and D. The next subdivision in the ClonalFrame tree (Figure 3A) separated JEA297p from the rest of clade 1, whereas the gene-content tree (Figure 3B) further divided the phylogeny into four subclades. One of these four subclades contained three of the four pathogenic isolates (JEA124p, JEA179p and JEA297p) despite the distant phylogenetic relationships among these (Figure 3A). This clade also included a commensal strain (EDM116c) which was not closely related to the pathogens according to the ClonalFrame core phylogeny, but contained a pathogenicity island and some of the genetic profile of a pathogen (Additional file 1: Table S1).

**Figure 4:**
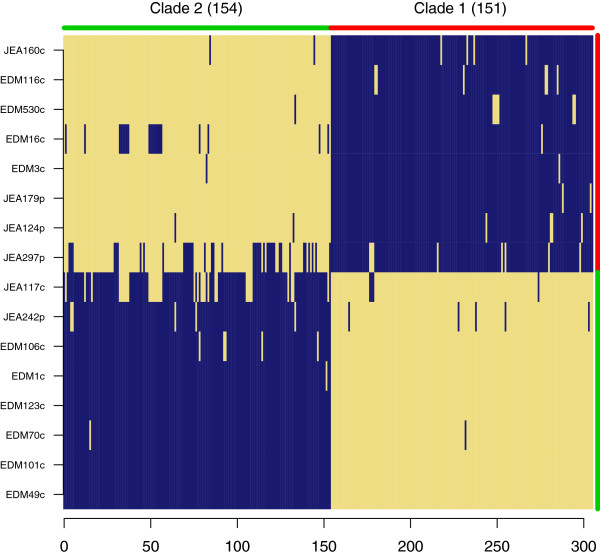
**Gene content enrichment comparing main clades 1 and 2.** Enrichment analysis was carried out using criteria I (Table 2). Both enrichments were highly significant (p > 0.001). The distribution of possible strain group permutations is presented in Additional file 1: Figure S2.

**Figure 5:**
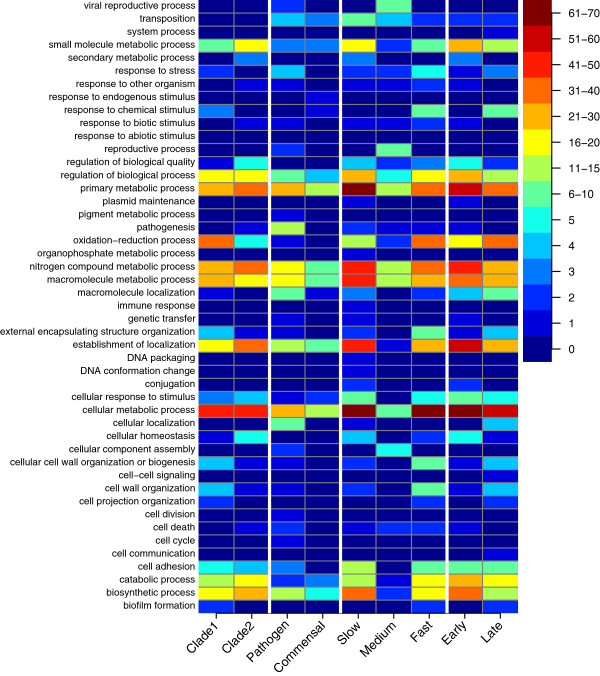
**General comparison of the enrichment profiles of the strain categories.** Each column is created from the gene enrichment list for each grouping (Additional file 16, Additional file 17, Additional file 18, Additional file 19, Additional file 20, Additional file 21, Additional file 22, Additional file 23 and Additional file 24). Each list of gene sequences was evaluated for ontology level 3 biological process categorization using Blast2GO for SEED assignments. The coloring scheme corresponds to enrichment scores assigned by Blast2GO. Grouping categories are shown on the x-axis, and the different comparisons are separated by white lines. Enrichment comparisons were performed between clade1 and clade2; pathogen and commensal; slow, medium and fast growth rates; early and late colonization. The color key indicates the enrichments scores for the biological processes.

Gene content enrichment analysis using the split between clades 1 and 2 to define the groupings (criteria I; Table 2) found 305 genes (Additional file 2: and Additional file 3) differentiating the clades with several of the gene sets falling into the same biological process categories (Figure 4, Figure 5). A relatively even distribution of genes (~150) were associated with each clade, and the level of enrichment of the clades was significantly higher than expected by chance (p < 0.001; permutation test; Additional file 1: Figure S2). Both clades are enriched for different cell adhesion proteins while clade 2 is differentially enriched for several additional iron acquisition proteins including an additional hemoglobin receptor, hemin transport protein, and yersiniabactin siderophore system. Clade 1 is differentially enriched for small molecule usage including an alternative pathway for obtaining nitrogen from cyanate, aromatic compound decomposition and resistance to potential toxins such as arsenic.

**Figure 6:**
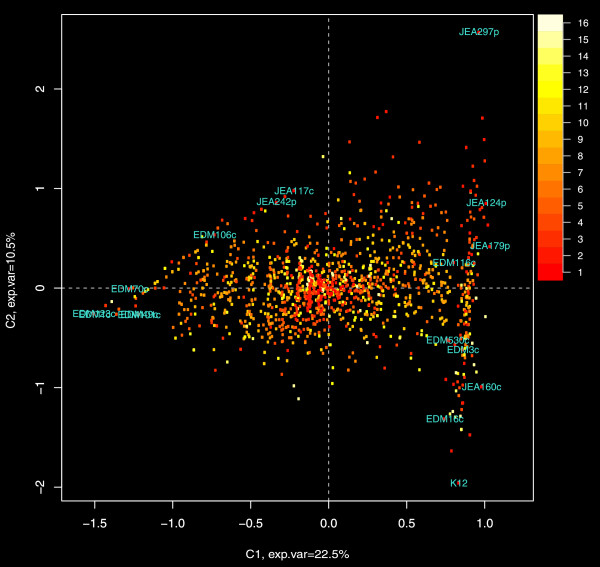
**Multiple correspondence analysis of the gene content matrix.** The plot shows principal coordinates along the two main components. Each point on the graph represents a gene and the color of the point relates the number of genomes in which it is present. The positions of the genome labels represent the relative distances of the genomes along the respective components.

**Table 2:** Criteria used for gene enrichment analyses

**Sorting criteria**	**Focal group (nr. strains)**	**Gene presence in focal group**	**Gene absence in non-focal group**
Criteria I, cladistic comparison	Clade1 (8)	≥7	≥7
	Clade2 (8)	≥7	≥7
Criteria II, pathogen/commensal comparison	Pathogen (4)	≥3	≥9
	Commensal (12)	≥9	≥3
Criteria III, growth rate comparison	Fast (2)	2	≥6
	Medium (4)	≥3	≥4
	Slow (4)	≥3	≥4
Criteria IV, colonization time comparison	Early (6)	≥5	≥4
	Late (6)	≥4	≥5
Criteria V, pathogen/commensal comparison	Pathogen (23)	≥17	≥13
	Commensal (17)	≥13	≥17
Criteria VI, pathogen/commensal comparison	Pathogen (5)	≥4	≥13
	Commensal (17)	≥13	≥4

**Criteria I**: *Criteria used for discriminating cladistic gene content enrichments. Each of the two clades contained 8 strains and enrichment required a gene to be present in at least 7 strains of one clade (focal group) while being absent from at least 7 strains in the other clade (non-focal group).*

**Criteria II**: *Criteria used for discriminating pathogen* vs. *commensal gene content enrichments. Since the two groups are of unequal size, a pathogen enriched gene had to present in at least 3 of 4 pathogenic strains and absent from at least 9 of 12 commensal strains. A commensal enriched gene had to be present in at least 9 of 12 commensal strains and absent from at least 3 of 4 pathogenic strains.*

**Criteria III**: *Criteria used for discriminating growth rate related gene content enrichments. The three growth rate categories (slow, medium and fast) contained 2,4, and 4 strains respectively. For a gene to be considered enriched in the fast category, a gene had to be preset in both fast strains and absent from at least 6 of 8 of the combined slow and medium strains. For a gene to be considered enriched in the medium category, a gene had to be preset in at least 3 of 4 medium strains and absent from at least 4 of 6 of the combined slow and fast strains. For a gene to be considered enriched in the slow category, a gene had to be present in at least 3 of 4 slow strains and absent from at least 4 of 6 of the combined medium and fast strains.*

**Criteria IV**: *Criteria used for discriminating early* vs. *late colonizer gene content enrichments. The two groups contain 6 strains each. Since one of the strains in the early group was also isolated in the late group (EDM123c). An asymmetrical enrichment profile was designed which required an early enriched gene to be present in at least 5 of 6 early strains and absent in at least 4 of 6 late strains. A gene enriched in the late colonizer group had to be present in at least 4 of 6 late strains and absent from at least 5 of 6 early strains.*

**Criteria V**: *Criteria used for discriminating pathogen* vs. *commensal gene content enrichments including 24 additional published E.coli genomes* (Table 3)*. Since the two groups are of unequal size, a pathogen enriched gene had to present in at least 17 of 23 pathogenic strains and absent from at least 13 of 17 commensal strains. A commensal enriched gene had to be present in at least 13 of 17 commensal strains and absent from at least 17 of 23 pathogenic strains.*

**Criteria VI**: *Criteria used for discriminating pathogen* vs. *commensal gene content enrichments including one additional published enteropathogenic E.coli (EPEC) genome and 5 additional published commensal isolate genomes* (Table 3)*. Since the two groups are of unequal size, a pathogen enriched gene had to present in at least 4 of 5 pathogenic strains and absent from at least 13 of 17 commensal strains. A commensal enriched gene had to be present in at least 13 of 17 commensal strains and absent from at least 4 of 5 pathogenic strains.*

### Pathogen and commensal comparisons

**Figure 7:**
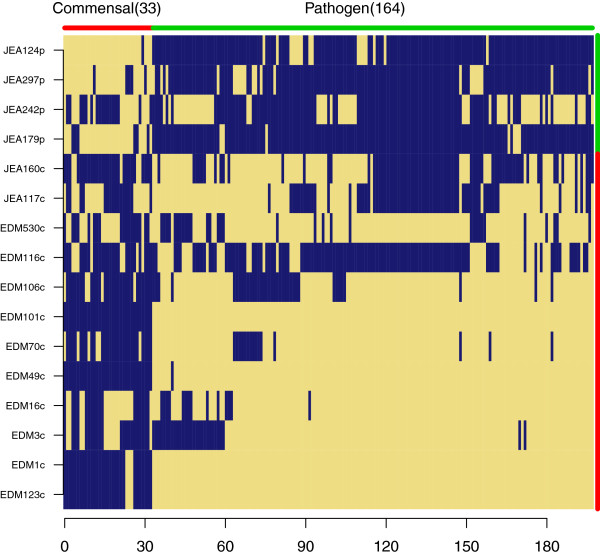
**Gene content profiles of pathogenic and commensal strains.** Enrichment analysis was carried out following criteria II (Table 2). 164 genes (p = 0.02) were found to be enriched in the pathogenic group while only 33 genes (p = 0.18) were enriched in the commensal group. The complete distributions of possible gene enrichments are presented in Additional file 1: Figure S3

Identifying the genetic elements that differentiate commensal and pathogenic strains is extremely important. Multiple correspondence analysis (see Materials and Methods) of the sequenced genomes highlighted differences between two groups of strains separated on the main axis of variation (Figure 6). The observed pattern matched the previously described cladistic structure (Figure 3), and showed a clustering of some of the pathogenic strains using an overall gene content similarity profile despite these strains being categorized into different phylogroups. We began by using the strictest criteria of group differentiation where a gene would have to be in 100% of the pathogenic strains and not found in the commensal group and vice versa. Surprisingly, this approach only identified a few chaperone genes. Relaxing the criteria (criteria II, Table 2) still yielded only 33 genes enriched in the commensal group but 164 in the pathogenic enrichment (Figure 7, Additional file 4 and Additional file 5). The probabilities of the commensal and pathogenic enrichments to happen by chance were equal to p = 0.18 and p = 0.02 respectively (permutation test; Additional file 1: Figure S3). Most of the commensal group gene enrichments were either related to fatty acid metabolism or sugar utilization pathways.

A noticeable contributor to the pathogen enrichment was the pathogenicity island carrying the type III secretion system (T3SS) and several effecter molecules associated with it. BLASTing the large contig sequences generated from the Newbler assemblies against a complete enterocyte effacement pathogenicity island (LEE) (35,624 bp) [15] revealed significant identity for many of the strains (Additional file 1: Table S1). All four pathogenic strains as well as three commensal isolates contained the pathogenicity island. Categories that were enriched in the pathogenic grouping relative to the commensal grouping included both nitrogen and primary metabolic processing (Figure 5). Not surprisingly, since the pathogenic strains were initially selected based on the presence of the intimin-encoding *eae* gene, and therefore belonged to pathotype enteropathogenic *E.coli* (EPEC) (Table 1), intimin enrichment was also observed in the pathogenic grouping.

### Growth rate comparisons

**Figure 8:**
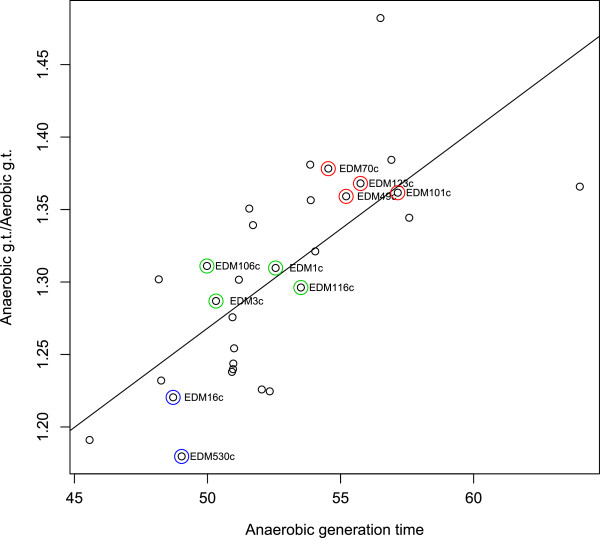
**Ratio of anaerobic/aerobic generation times related to anaerobic generation times of IMPACT isolates (de Muinck et al. submitted).** Circled strains are the ones for which we present genome sequences in this study. Blue circled strains are categorized as fast growers, green have a medium growth rate, and red circled strains are slow growing strains. R^2^ = 0.51, p < 0.0001.

**Figure 9:**
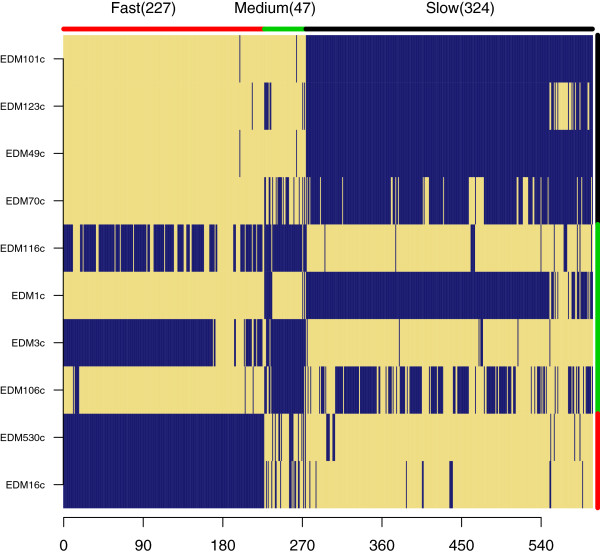
**Gene content profiles of slow, medium and fast growing strains.** Enrichment analysis was carried out using criteria III (Table 2). The fast category had 227 (p = 0.09) genes enriched. The medium growth rate category had only 46 (p = 0.56) genes enriched while the slow category had 324 (p = 0.04) genes. Distributions of possible enrichment profiles are shown in Additional file 1: Figure S4.

Plotting relationships between the anaerobic generation times and the ratio of anaerobic to aerobic generation times of the IMPACT isolates (Table 1) showed a strong positive correlation (Figure 8). Highlighting strains from which we have genome sequences showed three clusters that we then defined as fast, medium and slow growers. Evaluation of growth rate clustering, using a relatively complicated enrichment test due to the differences in numbers of strains in each of the groups (criteria III, Table 2), saw strong gene content distinction between the fast group (group of two) and slow group (group of four) with p-values of the gene content profile amounts equal to 0.09 and 0.04 respectively (Additional file 1: Figure S4). The strains with the medium growth rate (group of four) did not have a significant (p = 0.56) number of distinguishing genes. Enrichment profiles of the fast, medium and slow groups showed overrepresentation of 227, 47 and 324 gene families, respectively (Figure 9, Additional file 6, Additional file 7 and Additional file 8). Relative GO category enrichment in the slow group included primary metabolic process, nitrogen metabolism and macromolecular processes plus several genes important for iron uptake and utilization (Figure 5). The fast growing group was uniquely enriched for several GO categories including response to chemical stimuli and cell wall organization. The medium growth rate group seemed split between the slow and fast growers but the majority of genes enriched in this group were phage related.

### *Early* vs. *late colonizer comparisons*

**Figure 10:**
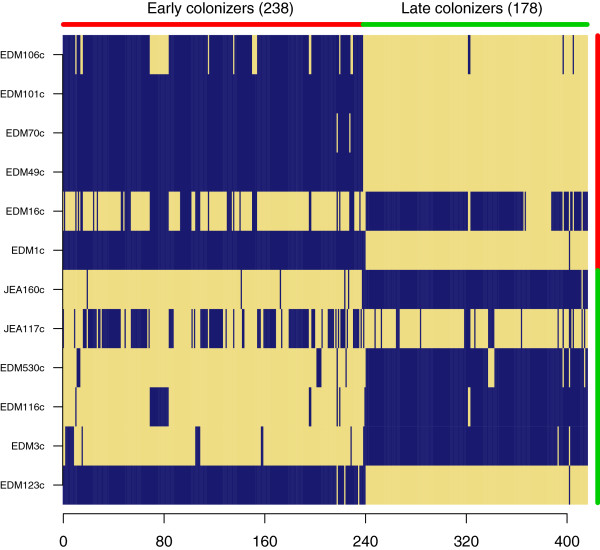
**Gene content enrichment profiles of early and late colonizer strains.** Enrichment analysis was carried out using criteria IV (Table 2). Both early and late colonizers show significant enrichments (p = 0.02 and p = 0.05, respectively). The complete distributions of possible enrichment profiles are shown in Additional file 1: Figure S5. EDM1c is an early colonizer that is clonally related to the late colonizer EDM123c. EDM123c maintains the early colonizer genomic profile but has lost genes found in the early colonizer profile.

In this collection of strains we categorized a strain as an early colonizer if the strain was isolated from an infant within the first two weeks of life. These strains have a higher likelihood of coming from the mother than isolates from later age categories [10]. Late colonizer strains were isolated from infants aged four months to two years. Comparison between the early colonizer and late colonizer strains found 416 genes that were differentially enriched between the two groups (criteria IV, Table 2, Figure 10, Additional file 9 and Additional file 10). One of the late colonizer strains was an early colonizer strain that had remained in the infant for four months (see below), this strain was considered an early colonizer and the enrichment criteria were modified to minimize bias. The 6 early colonizers and the 6 late colonizers had gene content profiles with p-values equal to 0.02 and 0.05 respectively (Additional file 1: Figure S5). Early colonizers were distinguished by 238 genes including capsular genes, fimbrial genes, yersiniabactin and other iron uptake systems as was also seen in the cladistic enrichment. Additionally, we found enrichment for type four pili, required for localized adherence and auto-aggregation phenotypes [16]. Late colonizer strains were distinguished by 178 genes including GO category biological enrichment for oxidation reduction processes and response to chemical stimuli (Figure 5).

### Codon usage bias and generation times

Codon usage bias in highly expressed genes has been found to be a strong predictor of maximal growth rate in prokaryotes [17]. In order to investigate this relationship in our data we looked at correlations between our effective number of codons (ENC) estimates and growth rates under aerobic and anaerobic conditions. Mean genome wide ENC for the 10 EDM strains was 49.044 ± 0.182, while mean ENC for ribosomal protein genes was 35.790 ± 0.052. Mean generation times were 40.3 ± 1.2 min. and 52.7 ± 3.0 min. under aerobic and anaerobic conditions, respectively. We first looked at the relationship between whole genome ENC and growth rate. We found a positive correlation (two-sided Spearman correlation ρ = 0.71, p-value = 0.03) with anaerobic growth rate (Additional file 1: Figure S6A), but no significant relationship with aerobic growth rate (ρ = 0.05, p-value = 0.89). This result indicated that faster growing isolates tend to have more pronounced overall codon bias. As expected the within species variation in ENC for ribosomal protein genes was minimal and no relationship could be found between this index and growth rates. Also due to a lack of variation in ribosomal protein ENC the relationships between ΔENC and growth rates were essentially the same as the genome wide correlations (ρ = 0.72, p-value = 0.02 and ρ = 0.26, p-value = 0.47 for anaerobic and aerobic generation times, respectively) (Additional file 1: Figure S6B).

### Strain evolution in the infant gut

Two pairs of strains from two infants (child 1891 and 1360, Table 1) were isolated at two different time points and had matching MLST profiles. The strains were isolated at four and eleven days of age (EDM49c and EDM101c) and at ten days and four months (EDM1c and EDM123c) of age respectively. The isolates from the same child had almost identical gene contents (Figure 2) and were subjected to closer scrutiny in order to shed light upon the selective pressures in a novel infant gut environment. The earlier isolate in each pair was thus defined as “the parent strain” and the later as “the evolved strain”.

Both gene content and codon usage indicated strain evolution in the infant gut from an early colonizer to late colonizer phenotype. From the EDM49c and EDM101c, only three genes, possibly phage related, were found in the parent strain but not in the evolved strain, and no genes were unique to the evolved strain relative to the parent strain. From EDM1c and EDM123c, 16 genes were found in the parent strain that were not in the evolved strain and 13 genes were found in the evolved strain not in the parent strain (Additional file 11 and Additional file 12). Interestingly, three of the genes unique to the parent strain were also called in the early colonization enrichment list whereas none of the genes unique to the parent strain were found in the list of genes from the late colonization enrichment. The three genes that were matched to the genes in the early enrichment list were GO categorized as a type-f conjugative transfer system pilin chaperone, hypothetical protein c4302 [uropathogenic, *E.coli* CFT073, NC_004431.1] and a tellurite resistance protein with transposon elements encoded nearby. Other genes unique to the evolved strain relative to the parent strain included a mercury resistance operon that has evidence of being carried on the transposon Tn21. Genome wide ENC and ΔENC comparison of EDM1c and EDM123c found reduced codon bias in the evolved strain (Additional file 1: Figure S6A and B).

### Relationship with other E.coli strains

**Figure 11:**
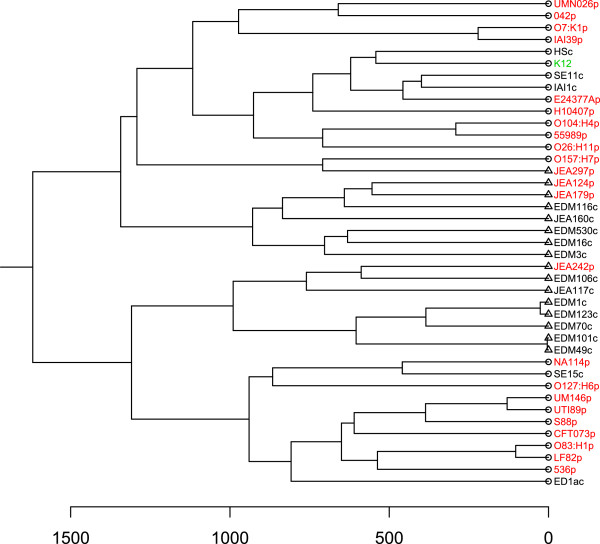
**Pan-genome tree of the 16 IMPACT isolates and 25 publicly available pathogenic and non-pathogenic isolates.** Pathogenic isolates are labeled in red, commensals are labeled in black, K12MG1655 is labeled in green. Leaves labeled with triangles represent strains genome sequenced as part of this study (Table 1). Leaves labeled with circles represent publicly available *E.coli* genomes downloaded from Genbank (Table 3). The pan-genome tree was created using hierarchical clustering of a Manhattan distance matrix based on the gene presence/absence matrix. The scale below the pan-genome tree indicates Manhattan distances.

In order to place the isolates described in this study into a wider context, the genomes sequenced as part of this study (Table 1) were compared with 25 previously published complete *E.coli* genome sequences (Table 3). The published genomes were selected to encompass a diversity of pathotypes and wild-type commensal strains. The addition of these 25 genomes lead to a 13.2% increase in pan-genome size, from 6152 to 6966 gene families, with a core genome of 2811 gene families (Additional file 1: Figure S8). A pan-genome tree was constructed using the gene presence/absence indicator matrix for these genomes and our 16 draft genomes (Figure 11). In this tree all our isolates, save JEA297p, occupied two sub-clades, each on one of the two main clades in the tree. Both of these sub-clades included commensals and pathogens, but no isolates originating from outside our study.

**Table 3:** List of publically available genomes used in gene content comparisons

**Nr.**	**Strain Name**	**Accession Number**	**Patho- type**	**Category**	**Reference**
1	K12MG1655	NC_000913.2		laboratory	[18]
2	O42	NC_017626.1	EAEC	pathogenic	[19]
3	536	NC_008253.1	UPEC	pathogenic	[20]
4	55989	NC_011748.1	EAEC	pathogenic	[5]
5	CFT073	NC_004431.1	UPEC	pathogenic	[21]
6	E24377A	NC_009801.1	ETEC	pathogenic	[22]
7	H10407	NC_017633.1	ETEC	pathogenic	[23]
8	HS	NC_009800.1		commensal	[22]
9	IAI1	NC_011741.1		commensal	[5]
10	IAI39	NC_011750.1	UPEC	pathogenic	[5]
11	LF82	NC_011993.1	AIEC	pathogenic	Direct Submission
12	UTI89	NC_007946.1	UPEC	pathogenic	[24]
13	UMNO26	NC_011751.1	UPEC	pathogenic	[5]
14	UMI46	NC_017632.1	AIEC	pathogenic	[25]
15	SE15	NC_013654.1		commensal	[26]
16	SE11	NC_011415.1		commensal	[27]
17	ED1a	NC_011745.1		commensal	[5]
18	S88	NC_011742.1	ExPEC	pathogenic	[5]
19	O157:H7	NC_002695.1	EHEC	pathogenic	[28]
20	O127:H5	NC_011601.1	EPEC	pathogenic	[29]
21	O104:H4	NC_018658.1	EHEC	pathogenic	[30]
22	O83:H1	NC_017634.1	AIEC	pathogenic	[31]
23	O26:H11	NC_013361.1	EHEC	pathogenic	[32]
24	O7:K1	NC_017646.1	ExPEC	pathogenic	[33]
25	NA114	NC_017644.1	UPEC	pathogenic	Direct Submission

EAEC – enteroaggregative *E.coli*, UPEC – uropathogenic *E.coli*, ETEC – enterotoxigenic *E.coli*, EPEC – enteropathogenic *E.coli*, AIEC – adherent-invasive *E.coli*, ExPEC – extraintestinal pathogenic *E.coli*, EHEC – enterohaemorrhagic *E.coli.*

We then conducted a gene content enrichment analysis in order to ascertain if there are general profiles that can distinguish pathogenic from commensal *E.coli*. Using the enrichment criteria listed in Table 2 (criteria V), we identified only 17 genes that were enriched in the commensals and none in the pathogens. 16 of the 17 commensal enriched gene families were short (<250 bp) sequences encoding hypothetical proteins and all were preferentially found in the strains sequenced in this study.

**Figure 12:**
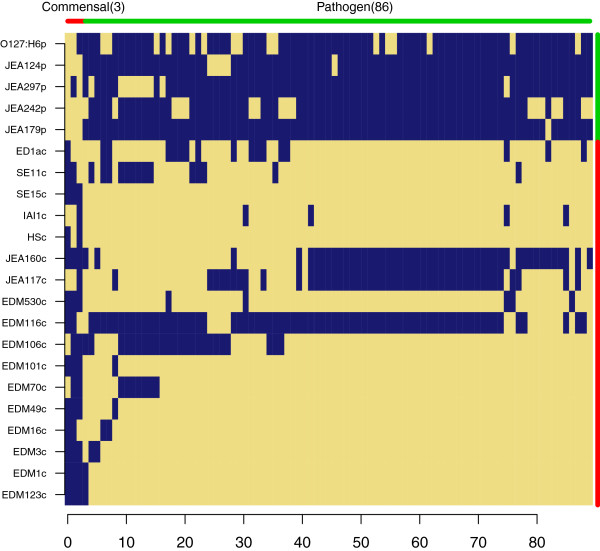
**Gene content profiles of enteropathogenic (EPEC) and commensal strains.** This enrichment analysis includes 6 additional genomes, 5 commensals and 1 EPEC, downloaded from Genbank (Table 3). Enrichment analysis was carried out following criteria VI (Table 2). 86 genes (p < 0.01) were found to be enriched in the pathogenic group while only 3 genes (p = 0.35) were enriched in the commensal group. The complete distributions of possible gene enrichments are presented in Additional file 1: Figure S9.

We focused and expanded the pathogen/commensal gene content comparison within our strains by including an additional publicly available complete genome sequence from an isolate categorized as EPEC which is the same pathotype as the four pathogens described in Table 1. We also expanded the commensal group with five complete genomes of wild-type commensal *E.coli* from Genbank. The enrichment criteria for this analysis are listed in Table 2 (criteria VI). This comparison found only three gene families enriched in the commensal group, but the EPEC group had a set of 86 enriched gene families (Figure 12, Additional file 13). While this is a lower number of pathogen enriched gene families than was found when including only the IMPACT strains in the analysis (Figure 7), it still represents a highly significant gene set (p < 0.01, Additional file 1: Figure S9).

## Discussion

### Genome analysis methods

The methodological challenges we addressed in order to generate the genotype-phenotype profiles presented in this work require some discussion. The 454 pyrosequencing single-end shotgun data presented difficulties that would, in several cases, not have been ameliorated by increasing the sequencing coverage (Additional file 1: Figure S1D). This is partly due to the intrinsic variability of *E.coli* genomic content, which made it impossible to rely on reference-based assembly and necessitated the use of *de novo* assembly methods, and also because of the relatively error prone nature of the technology. Alternative sequencing technologies or laborious and costly paired-end/mate-pair DNA sample preparation would have been required to reduce the number of contigs. However, the single-end shotgun approach offers a number of advantages due to its simplicity and lower cost compared with paired-end library preparation [34]. Furthermore, even though improvements in sequencing technologies will help genome assembly of bacterial isolates due to increased read length, sequencing of complex mixtures of bacteria such as gut or soil communities will continue to face some of the same challenges that we have addressed. The additional post-annotation search step employed in this study appears to have alleviated some of the biases introduced by an imperfect assembly (Additional file 1: Figure S1).

### *Pathogens* vs. *commensals*

The factors that distinguish a pathogenic from a commensal *E.coli* remain contentious. Previous studies have failed to come up with pathotype specific genomic cores for strains classified as enteropathogenic or enterotoxigenic *E.coli* (EPEC and ETEC, respectively) [22,23], but there have been studies reporting specific gene content profiles in extraintestinal pathogenic *E.coli* (ExPEC) [24,35]. However, recent work indicates that many of these genes are primarily associated with gut colonization and that virulence is an incidental by-product of commensalism [36,37]. In our case, using strict 100% presence/absence as an enrichment criterion failed to detect genes that separated commensals and pathogens (all four pathogenic strains were EPEC).

Relaxing the criteria resulted in a significant set of 164 genes that were preferentially found in the pathogenic group, but there was substantial gene overlap with commensal strains (Figure 7). The 33 genes enriched in the commensal group may represent a small part of the wide variety of genes necessary to be a successful colonizer. However, the weak commensal signature, compared with the pathogenic one, suggests that the term commensal may not be a meaningful descriptor in a phenotypic or evolutionary context as our analyses identified ‘pathogen-like’ commensals (e.g. commensal isolate EDM16c is closer to the pathogenic isolates when it comes to functional genetic profile than it is to the other commensals (Figures 3 &7)) which may suggest a virulence potential of certain commensal strains. This is especially highlighted by the large pathogenicity island carrying the TTSS which was shared by all the pathogenic strains and a subset of the commensals (Additional file 1: Table S1). Recent work has shown that this system is important for bacterial competition in the gut in addition to its role in host interactions [38]. If virulence is indeed an accidental by-product of adaptation to the gut environment it would explain why it is hard to find a non-clinical distinction between pathogenic and commensal strains, as virulence may rather be a matter of context and opportunism [39]. Genomic signatures may nevertheless identify strains that have greater capacity to make the transition from commensalism to virulence, and could thus aid in designing preventive strategies.

### Minimal generation time

Growth rate is a phenotype with quintessentially complex genetic underpinnings, and can hardly be ascribed to specific genes or alleles. Insight into the mechanisms underlying growth rate differences is highly desirable as it is related to other phenotypes of fundamental importance, such as virulence [40]. Minimal generation time in a study comparing 214 bacterial and archaeal species was found to correlate with genomic features such rRNA and tRNA copy number and codon usage bias [17]. However, minimal generation times were found to vary considerably within the *E.coli* isolates in our collection, even though these particular features were similar among our isolates.

We could not find any significant correlation between generation time and rRNA and tRNA copy number (results not shown), and codon usage bias was also found to be a poor predictor of aerobic generation time. Surprisingly, it correlated strongly with anaerobic generation time.

In contrast to the study by Vieira-Silva, we found a positive correlation between generation time and codon usage bias in highly expressed genes (ΔENC). This result is not necessarily in conflict with previous findings, as it may be explained by the fact that we were looking at strain level rather than species level relationships. Specifically, in contrast to the previous work covering many diverse species, the ribosomal protein genes were extremely conserved and the spread of ENC values for this set of sequences was less than a third of what was observed for genome wide ENC. Whole genome bias dominated our analysis and gave rise to the interpretation that a narrower general codon usage profile is associated with shorter anaerobic generation times. It is noteworthy that this relationship did not hold for aerobic growth. At face value it may seem paradoxical that codon usage specialization should be more important under anaerobic conditions when translation efficiency is presumably less of a limiting factor than under intrinsically faster aerobic growth. One explanation for this could be that gut adapted *E.coli* are primarily selected for anaerobic growth properties as the gut community matures and that aerobic growth leaves comparatively little systemic imprint on their genomes. Even though we found a significant correlation between aerobic and anaerobic generation time (R^2^ = 0.41, p < 0.001), we found and even stronger correlation between anaerobic generation time and anaerobic to aerobic generation time ratio (R^2^ = 0.51, p < 0.0001), suggesting that slow anaerobic growth entails disproportionally fast aerobic growth, and that the genomic bases for these two modes of growth might, at least in part, be uncoupled. This interpretation is supported by the fact that codon usage bias correlated with anaerobic but not aerobic growth rates. It would be interesting to compare these results with environmentally adapted *E.coli* isolates [6,41] and discern if the genomic imprint of aerobic growth might be more visible.

Gene content analysis represents an entirely different approach to investigating the genomic basis of differential generation time, and one that would not be appropriate for inter-species comparisons. The fact that our *E.coli* isolates are closely related, as witnessed by the shared genomic core, yet display relatively high variation in generation time begs the question of whether there are signatures of coding potential that relate to this phenotypic diversity. To our knowledge, the results presented here are the first attempt at correlating growth rate phenotype with differential gene content. Even though the small sample sizes warrant some caution in interpreting the results, the gene profiles of the fast and slow growing groups are quite unlikely to have arisen by chance. It is also noteworthy, albeit perhaps not surprising, that the intermediate group failed to produce a significant enrichment profile and that differences are only visible when comparing the extremes.

Relative enrichment in the slow group (324 genes) compared to the fast growing group (227 genes) found that many of the same GO categories were enriched but the slow growing group had a greater enrichment in several metabolic processes, including nitrogen, macromolecular, and several genes important for iron uptake and utilization (Figure 5). In contrast, the fast growers had a larger relative enrichment for genes involved in response to chemical stimuli and cell wall organization. Perhaps, this represents an ability to quickly adapt to changes in the environment. The fact that we observed relatively clear gene content signatures in both the fast and slow groups may reflect an evolutionary trade-off between short minimal generation time and scavenging potential. Copiotrophic, fast growing bacteria tend to have low affinity transporters typically representing an adaptation towards “feast” conditions, resulting in reduced competitiveness during nutrient starvation [42]. Slow growers, on the other hand, tend to have high affinity transporters, making them competitive in low nutrient environments, while at the same time making them susceptible to saturation or toxic effects when resources are plentiful [43]. This interpretation is further supported by the enhanced presence of scavenging-associated genes in our slow growing isolates.

### Early and late colonization

The infant gut environment is temporally dynamic in terms of reduction potential, nutrient availability, immune function and the structure of the resident microbial community [44,45]. The infant gut microbiome has been found to undergo a smooth increase in phylogenetic diversity over the first few years, while broad scale taxonomic patterns are characterized by abrupt events, eventually conforming to a mature profile [46]. The same study found concomitant changes in metagenomic content indicating that the community as a whole is responding to a changing environment. Selection pressures faced by members of the gut microbiota may therefore differ widely between the earlier and later stages of infancy. This pressure is reflected in the reduced relative abundance of *E.coli* in the mature microbiota relative to the infant gut community [45,47] and suggests that strains present at different stages of development could differ widely in their characteristics. Dramatic changes in the gut microbiota of pregnant women have also been shown from the first to third trimester, resulting in increased abundances of Proteobacteria and Actinobacteria and reduced taxonomic richness [48], a community state more reminiscent of the infant gut structure. The mother may somehow prime the gut microbiota with a qualitatively different environment in preparation for transfer to the infant.

Both early and late colonizers had significant differential gene content profiles (178 and 238 gene families respectively). We found that early colonizers were enriched for type IV secretion system and fimbrial genes that are important for attachment and interaction with the host. This group also had an increased presence of colicin resistance genes, which may reflect the importance of competition with bacteria of the same or closely related species is in the low diversity conditions of the early gut environment. Furthermore, we found an increased number of genes involved in biosynthetic processes in the early colonizer group. This could also be an adaptation to low diversity conditions where production of secondary metabolites and secreted growth factors is potentially limited. The late colonizers were enriched for resistance to toxins such as arsenate and cyanate. This could indicate the importance of these pathways for survival in the complex ecosystem of the mature gut.

### Evolution towards a late colonizer genomic profile

There is ample evidence that, given some selective regime, microbial evolution in the laboratory can be exceedingly rapid [49]. A few studies have documented the evolution of pathogenic bacteria in infected individuals [50,51] but reports of real-time evolution in natural environments remain scarce, and to our knowledge there are no such studies focusing on bacteria of the human gut. Isolate EDM123c was categorized as a late colonizer due to the fact that it was isolated from an infant at four months of age. EDM123c is by all probability clonally descended from EDM1c which had colonized that same infant already at 10 days after birth. Since this strain had spent nearly four months in the infant gut during an environmental transition period, we hypothesized that selection would push it toward a late colonizer genomic profile. There are two lines of evidence to suggest that this is the case. First, three of the genes that were present in the ancestral strain but lost from the evolved version matched genes in the early enrichment list. This list included a tellurite resistance protein which has been linked to resisting host defense [52,53]. Further experimentation is necessary to fully characterize the effect of these particular genes on early colonizing ability and possible reasons for negative selection in a more mature microbiota. Secondly, we observed an increased anaerobic generation time from isolate EDM1c (52.6 ± 0.4 min.) to EDM123c (55.8 ± 1.1 min.). Interestingly, EDM123c also had and elevated genome wide ENC (and thus also ΔENC) (Additional file 1: Figure S6) relative to the parent strain. This indicates that from the parent to the evolved strain there has been selection for synonymous mutations pushing the strain toward reduced codon usage bias. Reduced codon bias and growth rate have previously been associated with late gut colonization [17], indicating that isolate EDM123c has in fact evolved toward a late colonizer profile.

Given the close relatedness between EDM1c and EDM123C, as witnessed by both sequence similarity and gene content (Figure 3), there can be little doubt that these isolates are clonally related, and genomic differences are probably due to evolution taking place in the gut. Indeed the other pair of parental (EDM49c) and evolved (EDM101c) strains displayed practically no divergence in gene content or codon usage bias, probably due to the fact that they were isolated only 7 days apart. We cannot discount the possibility that clonally related strains were introduced, outcompeted and then re-introduced at a later time. In this case at least part of any evolution taken place would have done so in a different environment. In the case of EDM123c, however, we feel that this is an unlikely scenario since adaptation took the direction predicted if the isolate had evolved in a maturing infant gut.

### Cross category enrichment comparisons

Even though the different enrichment comparisons were fruitful for understanding functional categories, using this information across the different comparisons gave a better and more nuanced view. The main clade comparisons are very informative as they link a strain's evolutionary history to a measure of functional differentiation which can help define its ecological niche. For example, all early colonizers except EDM16c (which had an atypical gene content profile for an early colonizer) belong to clade2. The late colonizers all belong to clade1 except EDM123c, which is the evolved EDM1c and thus an atypical late colonizer. Thus there appears to be a phylogenetic split defining these ecological categories, and this split is reinforced by disparate gene content. Also, three of four pathogens group to clade1. Furthermore, and in contrast with the core genome phylogeny, the pan-genome phylogeny places the commensal strain EDM116c within the same subclade as these three pathogens (Figure 3). One could speculate that although EDM116c is an ostensibly asymptomatic isolate, its genetic makeup is such that given the right circumstances it may cause symptoms similar to known EPEC strains. The pathogenic isolate JEA242p, on the other hand, is placed within the otherwise exclusively commensal clade 2, demonstrating that virulence can emerge from quite different genomic backgrounds.

One of the two isolates classified as fast growing in this sample set of genome sequenced strains was a late colonizer (thus belonging to clade1) while early colonizers in this set tended to be slow growing (within clade2), but with disproportionally short aerobic relative to anaerobic generation times (Figure 8). This trend is not consistent with a previous study [17], but the disagreement is most likely attributable to sampling bias. Nevertheless some interesting associations emerged when making cross-grouping comparisons. Comparing the similarities in the gene content enrichments between all groupings found that the combined clade1-late-fast and clade2-early-slow designations shared the most (57 and 49 respectively; Additional file 14 and Additional file 15) (Additional file 1: Figure S7). Unique phosphotransferase systems (PTS) were enriched in each cross category grouping which are thought to enhance sugar utilization in general and possible bacterial uptake of sugars from breast milk [54]. A similar general differential gene content profile was seen between the same combined groups in glycosyl transferases and glycosyl hydrolase genes which are important for obtaining nutrients from the host and correct “assembly of a microbiota” [55]. The combined clade2-early-slow group further encoded arylsulfate sulfotransferase, which has been claimed to play a role in the detoxification of phenolic compounds [56]. On the other hand, a gamma aminobutyrate utilization gene was enriched in the combined clade1-late-fast group. This polyamine utilization gene has roles in proliferation under stressful conditions and utilization of alternative sources of carbon and nitrogen, which could be an adaptation to the difficult conditions of a mature gut microbiota [57,58]. Lastly, the clade1-late-fast group showed enrichment for the hydrogenase-4 operon, which is important in anaerobic growth [59]. These cross-category comparisons provide a tentative link between the evolutionary history and functional phenotypes of our isolates where the two main branches of the core and pan-genome phylogenies may represent adaptive paths leading toward distinctive ecological properties.

### Relationship with other E.coli strains

Expansion of the analysis to include published genome sequences reduced the core genome to 40.4%. This is rather high relative to previously reported values, but [3-5] considering the relatively close relatedness of the IMPACT strains (Figure 11) and the high degree of shared gene content (52.4%, Figure 1) among them, our result may not be so surprising. Methodological differences, e.g. cutoff values used for BLAST matching, will also have an impact on estimated core genome sizes.

The clustering of the IMPACT strains in the pan-genome tree constructed from all 41 genome sequences (Tables 1 and 3) illustrates the significance of geographical proximity in structuring of gene content profiles within the *E.coli* species. It is also evident from the pan-genome tree that pathogens and commensals are interspersed, and our general enrichment analysis confirmed that there were no significant gene sets discriminating pathogenic from commensal strains. This result highlights the genomic diversity within pathogenic *E.coli*, and that there are many different evolutionary pathways to pathogenicity. However, certain clades did seem to be dominated by either pathogens or commensals, but it is difficult to determine if this is due to sampling bias since most isolates in the commensal group are from the IMPACT study. Of note, the only IMPACT strain to group outside the two main IMPACT clades was JEA297p, a strain that stood out as having a low gene density relative to the other IMPACT isolates (Additional file 1: Figure S1F). This strain was located on a deep branch in separate clade shared only with the *E.coli* O157:H7 Sakai strain.

The lack of gene family enrichment observed in the general comparison between all 41 genomes is contrasted by the retention of an enrichment profile when the analysis is restricted to a single pathotype (Figure 12). The significant gene set that was retained in the enrichment analysis emphasized the importance of the LEE pathogenicity island components in defining EPEC strains (Additional file 13).

## Conclusions

This study addresses the role of gene repertoire in bacterial niche ecology, including the genomic bases of phenotypes that are not directly linked with pathogenicity. This aspect of *E.coli* ecology has not been thoroughly explored, but may shed light on the evolutionary history of the species [6]. The relatively small sample size and need for further molecular work precludes definitive conclusions regarding relationships between the array of genetic pathways and specific phenotypes. However, our results indicate a general pattern where alternative genetic pathways lead toward a consistent ecological role for *E.coli* as a species (Figure 5). Within this framework however, we saw selection shaping the coding repertoire of *E.coli* strains toward distinct ecotypes with different phenotypic properties. Additionally, the profiles we present should lead to further investigation and may lend insight into the biological roles of genes whose previously assigned biological function is incomplete and also for the large number of hypothetical proteins that were outlined using this method.

In contrast to previous studies of *E.coli* eco-genomics [3,5,22,60] our isolates come from a population that is narrowly localized both temporally and geographically. This could entail reduced genetic diversity due to shared ancestry and increased exchange of genes through horizontal transfer (HGT) between strains. Although the present study was not in particular concerned with HGT we did see a substantially higher percentage of shared gene content (52.4%) than what has previously been reported, as well as a smaller pan-genome, indicating that homogenizing forces are increasingly effecting genomic diversity on a local scale. This interpretation is also supported by the comparison of the isoloates sequenced in this study with publicly available *E.coli* genomes from a variety of sources (Figure 11), which showed that our strains were clearly distinguished from the others in terms of gene content. Nevertheless there were several instances where relatively clear gene enrichment profiles could be linked to specific phenotypes and ecological characters. Due to the disparate nature of *E.coli* genomes identification of such gene suites might be impeded if similar phenotypes can arise through different mechanisms and evolutionary histories, as is the case with clinical phenotypes of many pathogenic *E.coli*[5]. A more homogenous genomic background, as seen in this work, could make it easier to tease out gene content signatures that are ecologically relevant.

## Materials and methods

### Strains and culture conditions

The bacterial strains used in this study have been previously described in [10,14] and (de Muinck et al., manuscript submitted) (Table 1). 10 of these strains originated from samples collected as part of the IMPACT study [13]. Six strains were selected for genome sequencing from [14] because they were *eae*-positive and represented the previously reported diversity of phylogenetic groups. Two of these strains were from healthy children while four were isolated from children with diarrhea and these isolates were further classified as enteropathogenic *E.coli* (EPEC). A further ten strains were selected from [10], all of which were isolated from healthy children. All strains were grown to saturation in LB media and DNA extraction was performed using the DNeasy kit from Qiagen.

### Genome sequencing and annotation

DNA was single-end shotgun sequenced using Roche 454 GS (FLX Titanium) pyrosequencing. Sequences have been deposited in the EMBL-EBI Sequence Read Archive. Accession numbers are listed in Table 1. *De novo* assembly was performed using Roche's program Newbler v2.3 (performed at the freely available Bioportal computing service, http://www.bioportal.uio.no). Annotation of all genomes, including those downloaded from Genbank (Table 3), was done using RAST version 4.0 [61]. The RAST annotated genes of each of the genomes were BLASTed [62] against all the other annotated genomes using criteria of 85% identity and an e-value of less than 1x10^-25 to signify a gene match. Due to the large number of contigs, determination of gene presence included additional processing steps to recover genes split into separate contigs or genes that were not included in the annotation. Briefly, we used the complete set of annotated genes from all of the genomes as a reference pool. If a gene in the reference pool was not found in all of the analyzed genomes, the longest copy of the gene was re-BLASTed against the Newbler assemblies of each of the genomes in which the gene was initially not found. This gene was then added to the annotation of a genome if a partial hit was found that was at least 90% identical and an e-value of less than 1x10^-25. Genes were grouped as a family if they matched with the BLAST criteria just mentioned, or if they received identical functional annotations from RAST.

### Core genome phylogeny and pan-genome tree

A multiple alignment of the *de novo* genome assemblies was performed using progressiveMauve version 2.3.0 [63]. The regions shared by all genomes were then extracted and used to generate a phylogenetic tree using ClonalFrame version 1.2 [64]. In addition to this phylogeny based on the core genome, we constructed a tree based on the pan-genome as follows: a gene content matrix consisting of 1 s and 0 s was constructed where the columns correspond to the different strains and the rows to different gene families. An entry of 1 means presence of a gene family in a given strain, whereas a 0 means absence. This matrix was used for calculation of Manhattan distances between strains, which were then used for hierarchical clustering in order to construct the pan genome tree. These computations were done using R [65].

### Gene family enrichment analysis

Enrichment for gene families was found using the gene content matrix described above, combined with previous knowledge of the isolates. Isolates were grouped according to phenotypic or phylogenetic criteria and then gene families overrepresented in one group relative to others were counted in the matrix. Group sizes and cutoff values used to define overrepresentation are shown in Table 2. Results were plotted as heat maps in R. To assess the statistical significance of these results, we designed a permutation test in which we used the same group sizes as above but assigned group membership randomly according to a combinatorial scheme. This procedure produces the numerical distribution of gene family enrichments for all possible combinations of group members given some fixed set of group sizes and enrichment criteria, with which our results could be compared. This procedure provides an indication of whether our results could arise from random associations, although the limited strain sample means that subtle associations may go undetected. P-values for our focal enrichments were derived from the computed distributions as the empirical probability of observing an enrichment of equivalent or higher rank. Genes enriched in each of the groups and cross category comparisons are listed in Additional file 2, Additional file 3, Additional file 4, Additional file 5, Additional file 6, Additional file 7, Additional file 8, Additional file 9 and Additional file 10,Additional file 14 Additional file 15 and Additional file 13.

### Multiple correspondence analysis

Multiple correspondence analysis was carried out as described by Nenadic and Greenacre [66] using singular value decomposition of the scaled gene content indicator matrix.

### Enrichment for biological processes and re-annotation of enriched genes

The lists of genes generated by the gene family enrichment analysis and found to be overrepresented within each of the categories were used to generate the biological process scores using Blast2GO (http://www.Blast2GO.com) [67]. This software annotates coding sequences and assigns them to gene ontology (GO) categories. Blast2GO gene annotations of enriched and unique gene sets can be found in Additional file 11, Additional file 12, Additional file 16, Additional file 17, Additional file 18, Additional file 19, Additional file 20, Additional file 21, Additional file 22, Additional file 23 and Additional file 24.

### Codon usage bias analysis

Genome wide codon usage tables were computed from the annotated coding sequences for each strain. Codon usage for highly expressed genes was computed from the 54 ribosomal protein gene sequences extracted from the annotation of each EDM strain. Effective number of codons (ENC) was computed according to the method of Wright [68]. This provides a metric for the evenness of codon usage with smaller values indicating a bias toward more specialized codon usage while higher values signify more uniform usage. The index of bias in highly expressed genes, ΔENC, was computed as the scaled difference between genome wide ENC and highly expressed gene (ribosomal protein gene) ENC [17]. We did not apply correction for differential G + C content in our ENC calculations as this did not vary significantly across genomes.

## Abbreviations

BLAST: Basic local alignment search tool; ENC: Effective number of codons; EPEC: Enteropathogenic *E.coli*; GO: Gene ontology; HGT: Horizontal gene transfer; MLST: Multi-Locus Sequence Typing; PTS: Phosphotransferase system; RAST: Rapid annotations using subsystems technology.

## Competing interests

The authors declare that no competing interests exist.

## Authors’ contributions

Conceived and designed the experiments: ED and PT. Performed the experiments: ED and PT. Analyzed the data: ED, PT, KL, and XD. Contributed reagents/materials/analysis tools: JEA, NCS and XD. Wrote the paper: ED and PT (edits by KL, JEA, XD, KSR, KR, and NCS). All authors have read and approved the manuscript for publication.

## Supplementary Material

Additional file 1: Table S1 and Figures S1–S7. Click here for file

Additional file 2 Containing the RAST annotations of the individual enrichment analyses. Click here for file

Additional file 3 Containing the RAST annotations of the individual enrichment analyses. Click here for file

Additional file 4 Containing the RAST annotations of the individual enrichment analyses. Click here for file

Additional file 5 Containing the RAST annotations of the individual enrichment analyses. Click here for file

Additional file 6 Containing the RAST annotations of the individual enrichment analyses. Click here for file

Additional file 7 Containing the RAST annotations of the individual enrichment analyses. Click here for file

Additional file 8 Containing the RAST annotations of the individual enrichment analyses. Click here for file

Additional file 9 Containing the RAST annotations of the individual enrichment analyses. Click here for file

Additional file 10 Containing the RAST annotations of the individual enrichment analyses. Click here for file

Additional file 11 Containing blast2GO re-annotations unique genes in the parental strain (EDM1c) and evolved strain (EDM123c). Click here for file

Additional file 12 Containing blast2GO re-annotations unique genes in the parental strain (EDM1c) and evolved strain (EDM123c). Click here for file

Additional file 13 Containing RAST annotations of the enrichment analysis including an additional EPEC strain and 5 additional commensals. Click here for file

Additional file 14 Containing RAST annotations of the cross-category enrichments analyses. Click here for file

Additional file 15 Containing RAST annotations of the cross-category enrichments analyses. Click here for file

Additional file 16 Containing blast2GO re-annotations of the individual enrichment analyses. Click here for file

Additional file 17 Containing blast2GO re-annotations of the individual enrichment analyses. Click here for file

Additional file 18 Containing blast2GO re-annotations of the individual enrichment analyses. Click here for file

Additional file 19 Containing blast2GO re-annotations of the individual enrichment analyses. Click here for file

Additional file 20 Containing blast2GO re-annotations of the individual enrichment analyses. Click here for file

Additional file 21 Containing blast2GO re-annotations of the individual enrichment analyses. Click here for file

Additional file 22 Containing blast2GO re-annotations of the individual enrichment analyses. Click here for file

Additional file 23 Containing blast2GO re-annotations of the individual enrichment analyses. Click here for file

Additional file 24 Containing blast2GO re-annotations of the individual enrichment analyses. Click here for file
