# Supplementary material for: Comparisons of infant Escherichia coli isolates link genomic profiles with adaptation to the ecological niche
Source: BMC Genomics. 2013 Feb 5;14:81. doi: 10.1186/1471-2164-14-81 (PMC3637554; doi:10.1186/1471-2164-14-81)
Supplement: Additional file 1: Table S1 — and Figures S1–S7. [file 1471-2164-14-81-S1.docx]

**Supporting information: Supplementary table 1. Supplementary figures 1-9.**

**Comparisons of infant *Escherichia coli* isolates link**

**genomic profiles with adaptation to the ecological niche**

Eric J. de Muinck (ericdemuinck@gmail.com)^1,2,3^

Karin Lagesen (karin.lagesen@bio.uio.no)^1^

Jan Egil Afset (jan.afset@ntnu.no)^4,5^

Xavier Didelot (x.didelot@imperial.ac.uk)^6^

Kjersti S. Rønningen (kjersti.skjold.ronningen@gmail.com)^7^

Knut Rudi (knut.rudi@umb.no)^8^

Nils Chr. Stenseth (n.c.stenseth@bio.uio.no)^1^

Pål Trosvik, corresponding author, (pal.trosvik@bio.uio.no)^1^

1 Centre for Ecological and Evolutionary Synthesis (CEES), Department of Biology, University of Oslo, Po Box 1066, 0316 Oslo Norway

2 Division of Epidemiology, Norwegian Institute of Public Health, PO Box 4404, 0456 Oslo, Norway

3 NOFIMA - The Norwegian Institute of Food, Fisheries and Aquaculture Research, PO Box 210, 1430 Ås, Norway

4 Department of Laboratory Medicine, Children’s and Women’s Health, Faculty of Medicine, Norwegian University of Science and Technology, PO Box 8905, 7491 Trondheim, Norway

5 Department of Medical Microbiology , St Olavs Hospital, PO Box 3250, 7006 Trondheim, Norway

6 Department of Infectious Disease Epidemiology, Imperial College London, St Mary’s Campus, Norfolk Place, London W2 1PG, UK

7 Department of Pediatric Research, Oslo University Hospital, Rikshospitalet, PO Box 4905, 0424 Oslo, Norway

8 Department of Chemistry, Biotechnology and Food Science, University of Life Sciences, PO Box 5003, 1432 Ås, Norway

**Table S1:** Evaluation of genome sequenced strains for the presence of the enterocyte effacement pathogenicity island. The complete 35,624 base pair LEE pathogenicity island of *E.coli* strain E2348/69 (AF022236.1) was BLASTed against each of the genome assemblies. Strains not included in the table showed no significant identity. The lengths of the sequences found in the genome of each strain with greater than 90% identity were summed and the percent of the summed length was compared with the complete 35,624 base pair length of the pathogenicity island.

|  | percent of total |
| --- | --- |
|  | alignment length |
| Strain ID | >90% identity |
| EDM116c | 98% |
| JEA117c | 96% |
| JEA160c | 94% |
| JEA179p | 93% |
| JEA242p | 99% |
| JEA297p | 74%,88%* |
| JEA124p | 49%,88%* |
|  | *80%identity |
|  | threshold value |

*the same comparison was repeated for some strains using a reduced identity threshold of 80%.


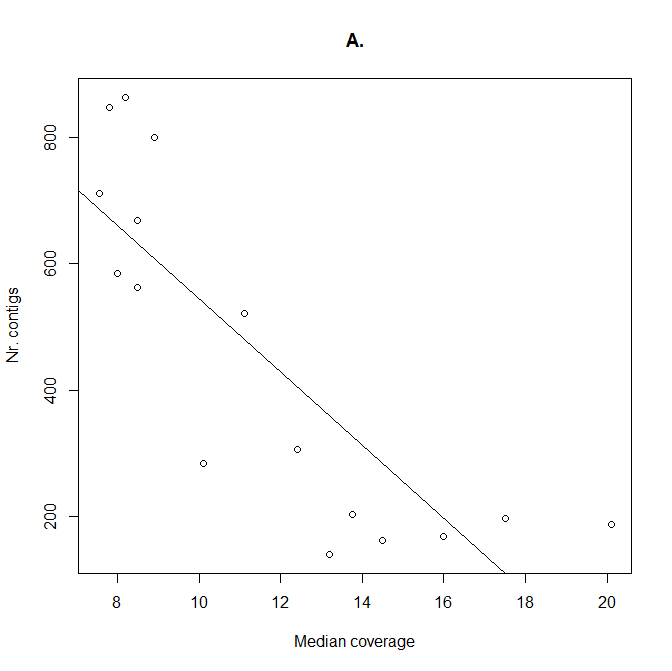

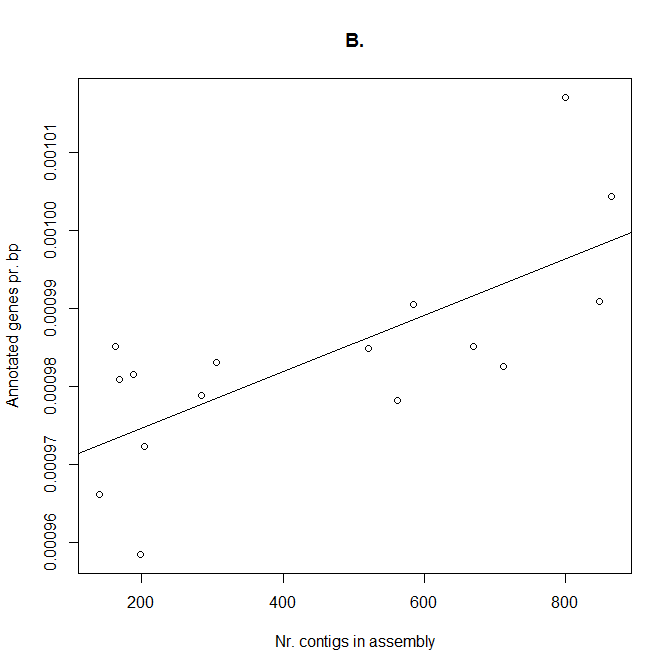


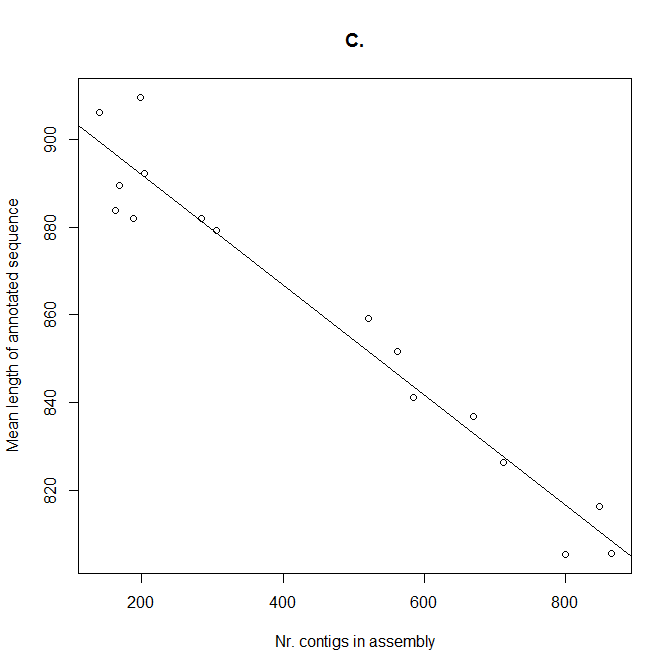

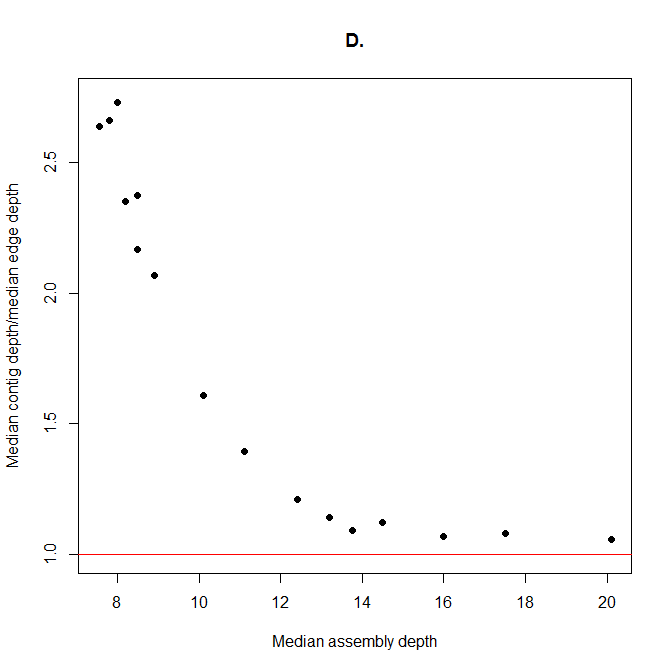


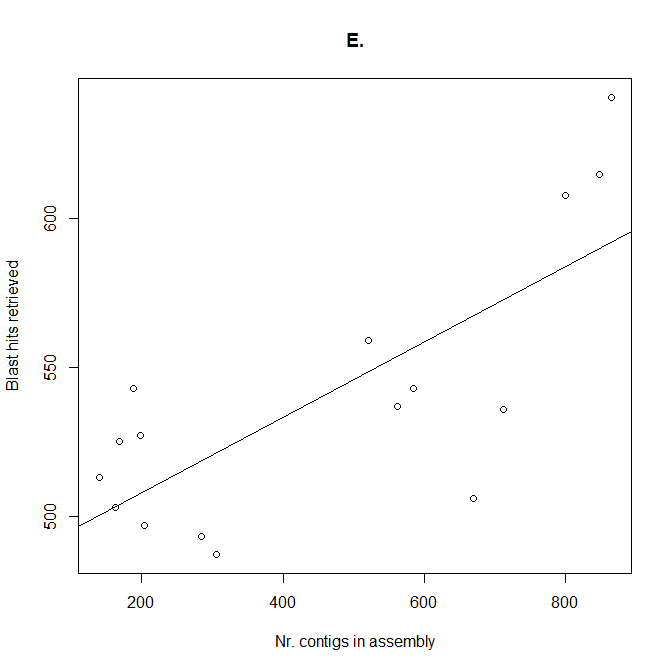

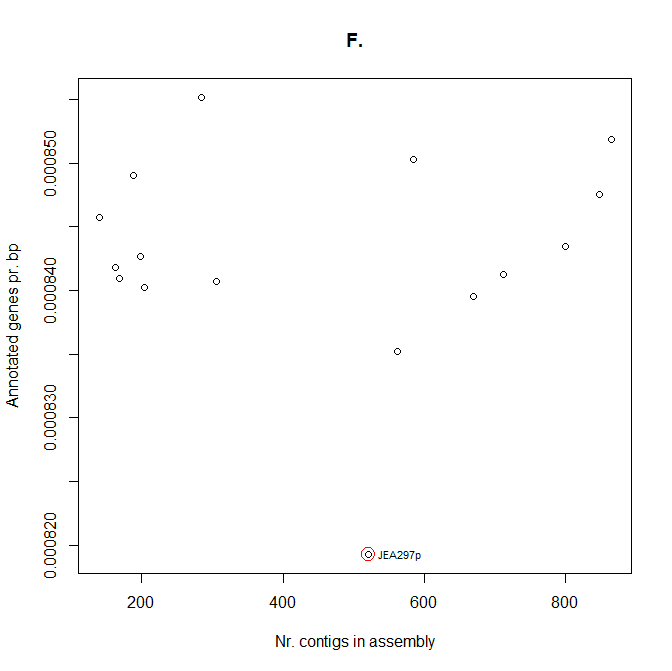


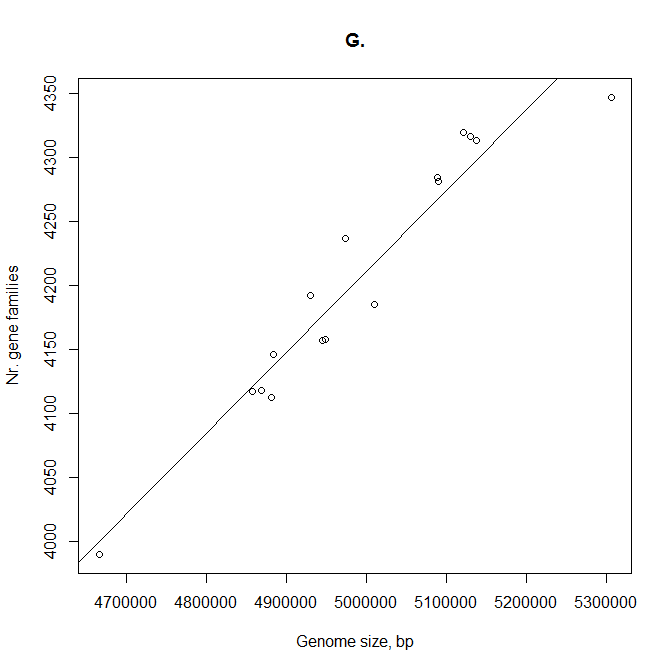


**Figure S1**: **A**. Decrease in the number of contigs in an assembly as the depth of coverage increases (R^2^=0.69, p<0.0001). **B.** Relationship between the number of contigs in an assembly and the number of annotated sequences (R^2^=0.52, p=0.0017). **C.** Decrease in the average annotated sequence length relative to the number of contigs in an assembly (R^2^=0.94, p<0.0001). **D**. Relationship between the median assembly read depth and the ratio of the median depth of the contigs to the median depth of the contig edges. Edge contig read depths were estimated from the outermost 1% of the total length on either side of all contigs of at least 1000 base pairs within each assembly. **E.** Relationship between the number of contigs in an assembly and the number of partial genes retrieved from re-BLASTing annotated sequences against the complete genome assemblies (R^2^=0.57, p=0.0008). **F**. Relationship between coverage depth and number of genes after the additional processing steps (R^2^<0.0001, p=0.97). **G.** Correlation between the number of gene families and genome size (R^2^=0.92, p<0.0001).


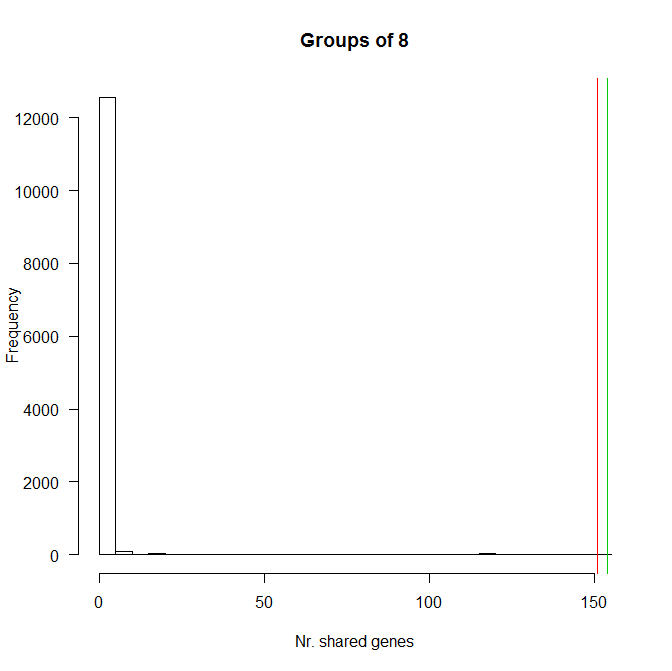


**Figure S2:** Distribution of possible gene content enrichment profiles using the cladistic enrichment criteria (criteria I, Table 2). ‘Groups of 8’ describes the number of strains in each category and the numbers of shared genes are shown on the bottom axis. The red and green lines show the number of shared genes in clade1 (151) and clade2 respectively (154). The cladistic grouping had the most significant (p<0.0001) distribution of the tested categories.


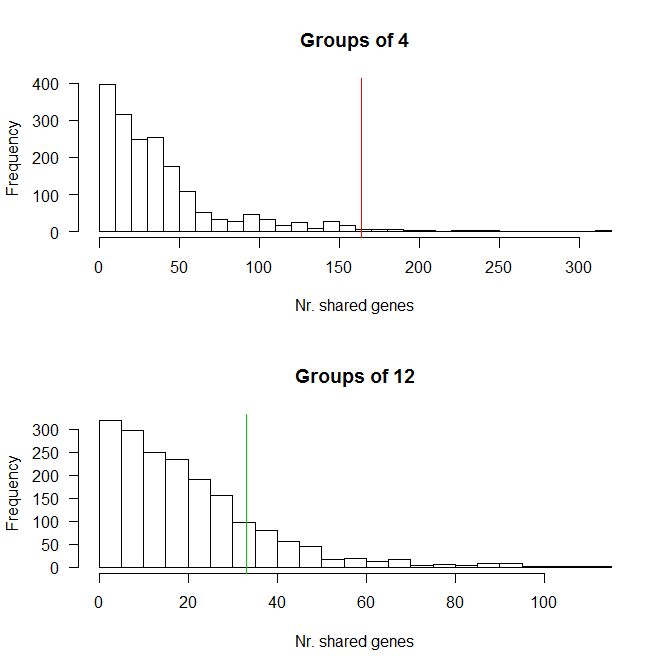


**Figure S3:** Distribution of possible gene content enrichment profiles using permutations of the groupings described by criteria II (Table2). The number of genes in an enrichment profile is shown on the y-axis. ‘Groups of 4’ corresponds to the sorting criteria used for pathogens (red line). ‘Groups of 12’ corresponds to the sorting criteria for the commensals (green line).


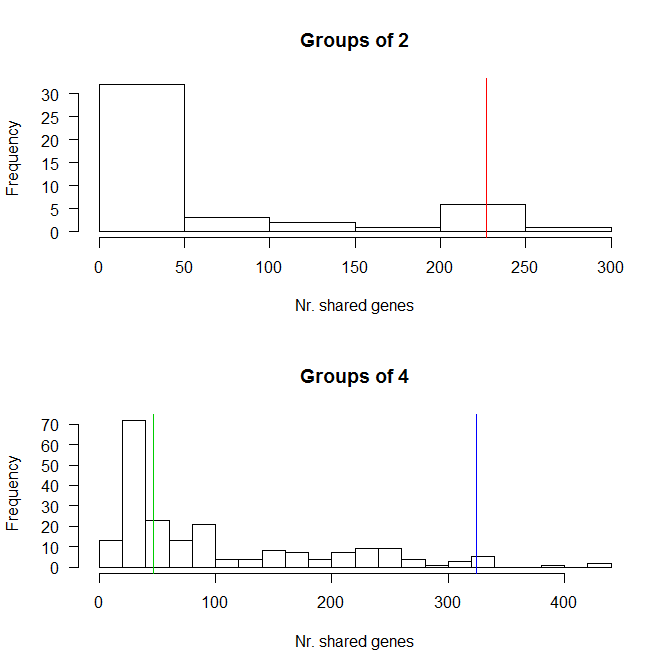


**Figure S4:** Distribution of possible gene content enrichment profiles of the growth rate groupings described by criteria III (Table2). ‘Groups of 2’ corresponds to the sorting criteria used for fast growers (red line). ‘Groups of 4’ corresponds to the sorting criteria used for the medium (green line) and slow growers (blue line).


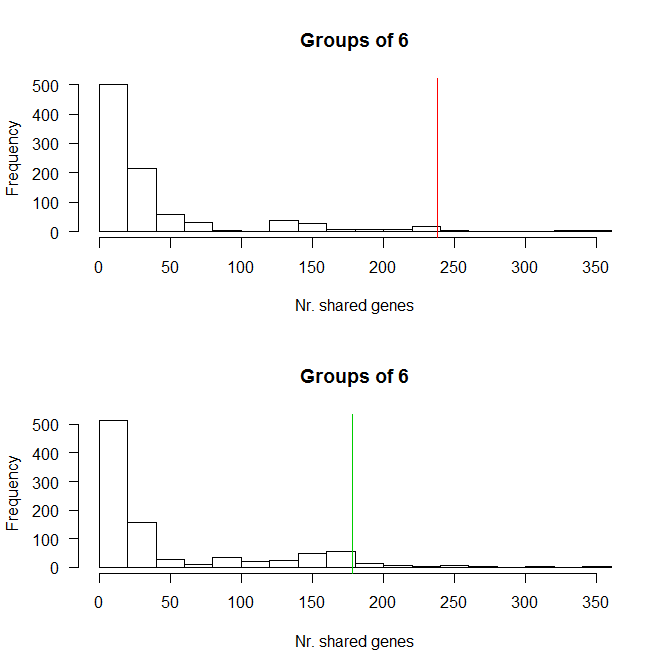


**Figure S5:** Distribution of possible gene content enrichment profiles of the early and late colonizer groupings described by criteria IV (Table2). The top panel ‘Groups of 6’ corresponds to the sorting criteria used for early colonizers (red line). The bottom panel ‘Groups of 6’ corresponds to the sorting criteria used for the late colonizers (green line). The use of two panels is due to the different distributions produced by the asymmetric sorting criteria used for the two gene content enrichment categories.

**
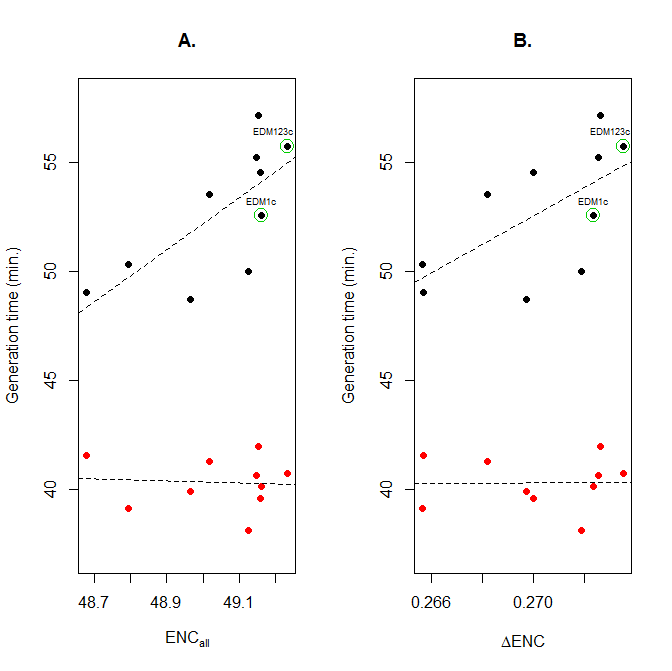
Figure S6:** Plots of codon usage bias vs. aerobic (red dots) and anaerobic (black dots) generation times. **A.** Genome wide codon usage bias (ENC_all_). **B.** Codon usage bias in highly expressed genes (ΔENC), represented by 54 ribosomal protein genes. Dashed lines are linear regression fits. The parent (EDM1c) and evolved (EDM123c) isolates separated by 4 months are marked with green rings.

**
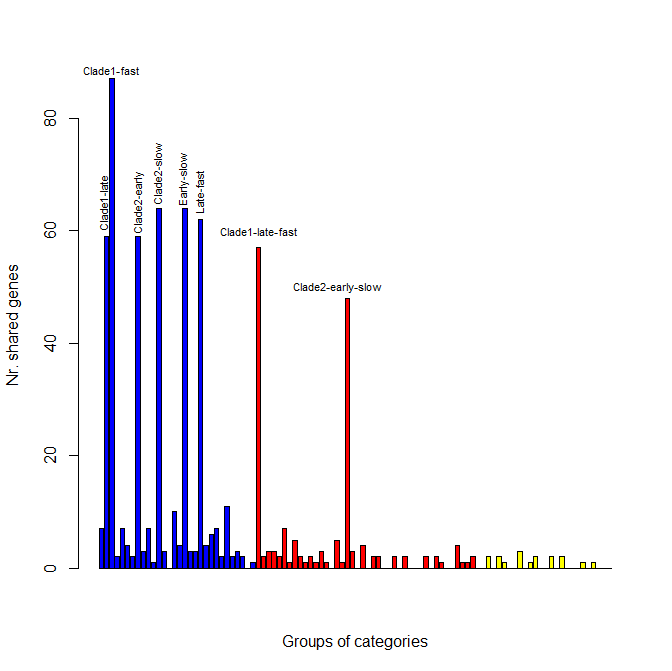
Figure S7:** Number of genes enriched across grouping categories. All possible comparisons between main clade provenance, time of colonization, growth rate and pathogenicity are represented on the x-axis with selected outcomes labelled above the bars. The y-axis shows the number of Blast2GO annotated genes that are common to two or more categories. Blue bars are pairwise comparisons. Red bars are three-way comparisons. Yellow bars are four-way comparisons.

**
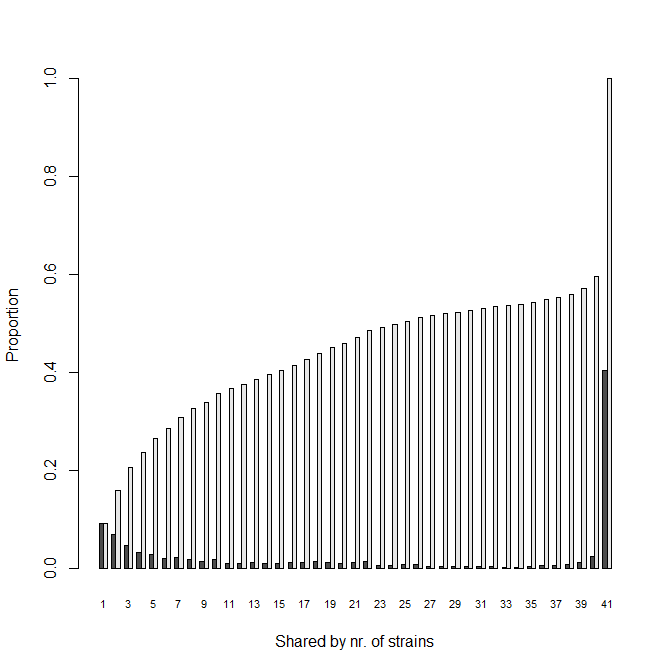
Figure S8:** Relative and cumulative proportions of genes as the number of included genomes increases. Each pair of bars along the x-axis represents the relative (dark grey) and cumulative (light grey) proportions of genes that are shared among the indicated number of strains. E.g. the first pair of bars indicates the proportion of genes that were found in only one of the 41 strains, the second pair indicates the proportion shared by two of the 41 strains, while the final pair indicates the proportion of the pan-genome that is common to all 41 strains. All duplicated gene annotations were removed for this analysis. 9.1% of annotated genes are unique to one strain while 40.4% are common to all. The total number of gene families in the pan-genome is 6966.


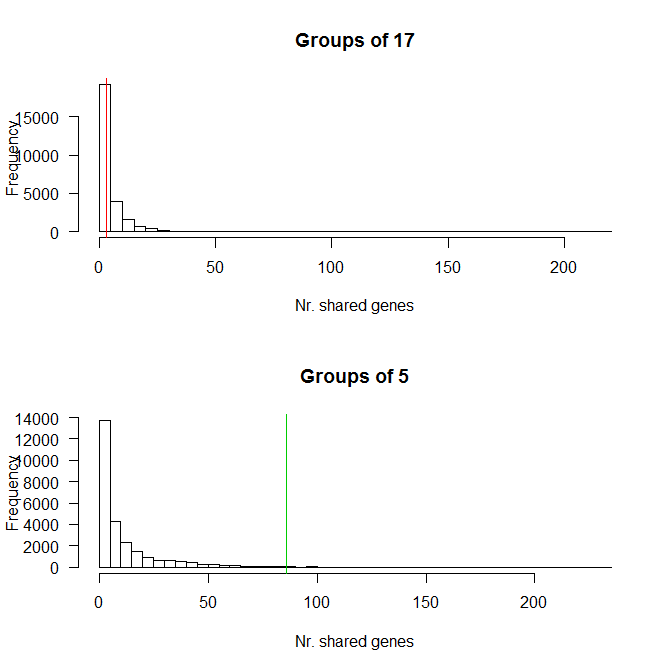
**Figure S9:** Distribution of possible gene content enrichment profiles using permutations of the groupings described by criteria VI (Table2). The number of genes in an enrichment profile is shown on the y-axis. ‘Groups of 17’ corresponds to the sorting criteria used for commensals (red line). ‘Groups of 5’ corresponds to the sorting criteria for the pathogens (green line).
